# Associations of Fasting Blood Glucose with Influencing Factors in Northeast China: A Quantile Regression Analysis

**DOI:** 10.3390/ijerph14111368

**Published:** 2017-11-10

**Authors:** Xin Guo, Li Shen, Jing Dou, Yaogai Lv, Anning Zhang, Fanchao Shi, Zhiqiang Xue, Yaqin Yu, Lina Jin, Yan Yao

**Affiliations:** Epidemiology and Statistics, School of Public Health, Jilin University, Changchun 130021, China; gggxxxfly@163.com (X.G.); shenli16@mails.jlu.edu.cn (L.S.); doujingjiayou@163.com (J.D.); lvyg16@mails.jlu.edu.cn (Y.L.); zhangan16@mails.jlu.edu.cn (A.Z.); sfc82123187@163.com (F.S.); xuezq16@mails.jlu.edu.cn (Z.X.); yuyq@jlu.edu.cn (Y.Y.)

**Keywords:** diabetes mellitus, lifestyle, quantile regression

## Abstract

*Background*: Diabetes mellitus (DM) has become a major public health problem in China. Although a number of researchers have investigated DM risk factors, little is known about the associations between values of fasting blood glucose (FBG) and influencing factors. This study aims to explore these associations by the quantile regression (QR) model. *Methods*: A cross-sectional survey based on a sample of 23,050 adults aged 18 to 79 years was conducted in Jilin in 2012, and some subjects were excluded due to missing values with respect to necessary variables or having glycemic control, in accordance with the purposes of this study. Finally, in total 14,698 people were included in this study. QR was performed to identify the factors influencing the level of FBG in different quantiles of FBG. *Results:* The distribution of FBG status was different between males and females (*χ*^2^ = 175.30, *p* < 0.001). The QR model provided more detailed views on the associations of FBG with different factors and revealed apparent quantile-related patterns separately for different factors. Body mass index (BMI) was positively associated with the low and middle quantiles of FBG. Waist circumference (WC) had a positive association with the high quantiles of FBG. *Conclusions*: FBG had a positive association with BMI in normal FBG, and a positive association with WC in high FBG. Diet and alcohol intake were associated with FBG in normal FBG. FBG was more likely to be elevated in the elderly, female workers, and people with family history of DM.

## 1. Introduction

Diabetes mellitus (DM) is currently one of the most important public health problems in the world, and the prevalence of DM and DM-related deaths is rising sharply [1,2,3]. The International Diabetes Federation (IDF) estimated that the number of people with DM was around 382 million worldwide in 2013, of which almost one-third were in China [4]. Furthermore, DM not only affects the patients’ quality of life, but also brings heavy economic burden to individuals, families and society [5].

As the number of studies on DM is growing, many DM-related risk factors have been recognized as well. A number of studies showed that obesity, poor eating habits, and physical inactivity could increase the prevalence and case fatality rate of DM, with males and the elderly being at greatest risk. Most studies investigated DM as a categorical variable [6,7,8,9,10]. The occurrence and development of DM is a continuous, long-term process, while fasting blood glucose (FBG) is a sensitivity index for diagnosing DM which reflects the progress of the disease to a certain extent. Some studies have investigated the effects of different levels of FBG on human health. A number of studies have shown that elevated FBG implies a higher risk of worsening illness [11,12]. Kosiborod et al. [13] documented that the association between average FBG during hospitalization and all-cause mortality rate was a ‘‘J-shaped’’ curve in a study of 16,871 patients with acute myocardial infarction. Moreover, fasting serum uric acid levels were higher in the pre-diabetic population, but lower in non-diabetic and diabetic patients [14]. That is to say all levels of FBG (not only in DM) have health significance, but there still exists a huge gap in our understanding of the levels of FBG.

Ordinary least squares (OLS) regression only describes the general information on average, which cannot fully reflect the information of the overall distribution. For example, populations with low weight (rather than average weight only) have been the focus in neonatal studies. Besides, when modelling data with heterogeneous conditional distributions, OLS results in estimation bias [15]. Quantile regression (QR), which was firstly proposed by Koenker and Bassett in 1978, can generalize OLS, and the weighted least absolute value method has been used in estimations [16]. Meanwhile, QR can build a series of regression equations in all quantiles without extra hypotheses in distributions. Hence, QR was applied to analyze extreme area data, such as low and high quantiles of FBG in the present study [17].

In this study, we explored and identified how the levels of FBG were associated with influencing factors using a multivariate QR model. Participants were part of the study on the prevalence of chronic disease and risk factors among adults in Jilin Province, northeast China (latitude 40°–46°, longitude 121°–131°) in 2012. We found that FBG had a positive association with body mass index (BMI) in normal FBG, whereas FBG had a positive association with waist circumference (WC) in high FBG. Therefore, we could develop more effective prevention strategies for different FBG populations, so as to achieve the purpose of “precision prevention”.

## 2. Materials and Methods

### 2.1. Study Population

Data was derived from a cross-sectional study of chronic disease conducted by School of Public Health, Jilin University, and the Jilin Department of Health in the Jilin Province of China in 2012. In total, 23,050 participants who had lived in Jilin Province for more than 6 months and who were aged between 18 and 79 years old were selected through multistage stratified random cluster sampling [18] (see details in Part 1 of the Supplementary Materials). In this study, we aimed to investigate the associations between FBG and influencing factors. For this purpose, some subjects were excluded due to missing values in FBG (6220 subjects) or having glycemic control (2132 subjects). In total, of 14,698 people were included in this study.

### 2.2. Ethics Statement

The ethics committee of the School of Public Health, Jilin University (Reference Number: 2012-R-011) and the Bureau of Public Health of Jilin Province (Reference Number: 2012-10) approved the study, and written informed consent was obtained from all of the participants before data collection.

### 2.3. Data Collection and Measurements

All information was collected by investigators who had been uniformly trained. The data included demographics (e.g., gender, age, occupation, etc.), health-related behaviors (e.g., smoking, drinking, etc.), dietary habits (e.g., vegetable, fruit, meat, etc.), and anthropometric measurements (e.g., height, weight, FBG, etc.).

FBG was measured by the Bayer Bai Ankang fingertip blood glucose monitor machine (Bayer, Leverkusen, Germany) and serum lipids levels (triglyceride (TG), total cholesterol (TC), low-density lipoprotein cholesterol (LDL-c) and high-density lipoprotein cholesterol (HDL-c)) were measured by the MODULE P800 biochemical analysis machine (Roche Co., Ltd., Shanghai, China) in the morning after participants fasted for 10 or more hours overnight. The participants’ height, weight and waist circumference (WC) were measured though a standardized protocol and process, wearing clothing but no shoes. Body mass index (BMI) was calculated by the following formula: BMI = Weight (kg)/Height (m^2^).

### 2.4. Assessment Criteria

DM was defined as FBG ≥ 7.0 mmol/L; impaired fasting glycaemia (IFG) was defined as FBG < 7.0 mmol/L and FBG ≥ 6.1 mmol/L; euglycemia was defined as FBG < 6.1 mmol/L and FBG ≥ 3.9 mmol/L; and hypoglycemia was defined as FBG < 3.9 mmol/L [19,20]. Manual labor occupations included production workers, farmers, and service workers. Mental labor occupations included office and other technical staff. Other occupations included students, the unemployed and retirees. Smokers were defined as persons who had smoked more than one cigarette per day in the past 30 days. Drinkers were defined as persons who had consumed an average of more than one alcoholic drink per week in the past 30 days, including spirits, beer, wine or other forms of alcohol. Dietary habits were classified into two types: “occasionally/rarely” and “often”. “Occasionally/rarely” was defined as consumption of less than three times per week. “Often” was defined as consumption three times per week or more. Exercise was also classified into two types: “occasionally/rarely” was defined exercise performed seldom or never. “Often” was defined as exercise three times per week or more with a duration of each exercise more than 30 min [18].

### 2.5. Statistical Analysis

The continuous variables were presented as the means ± standard deviations (SD) and median (inter-quartile range, IQR), compared using Student’s *t*-test or rank-sum test. The categorical variables were presented as counts or percentages and compared using the Rao–Scott chi-square test. Multivariate QR was performed to identify the factors which influenced the FBG level in different quantiles of FBG. Statistical analysis was performed using the R version 3.3.3 (R Foundation for Statistical Computing, Vienna, Austria) and the package “quantreg” [21,22]. Statistical significance was set at *p*-value < 0.05.

## 3. Results

As shown in Table 1, BMI, WC, FBG, and TG were significantly higher in males than those in females (*p* < 0.05). However, age and LDL-c and HDL-c levels were significantly higher in females than in males (*p* < 0.05). Then, there were differences between males and females in occupations, smoking, drinking, family history of DM, dietary habits (vegetable, fruit, meat, fish, eggs/bean/bean products, milk/dairy products) and exercise (*p* < 0.05). Meanwhile, residence and TC did not differ significantly by gender (*p* < 0.05).

**Figure 1:**
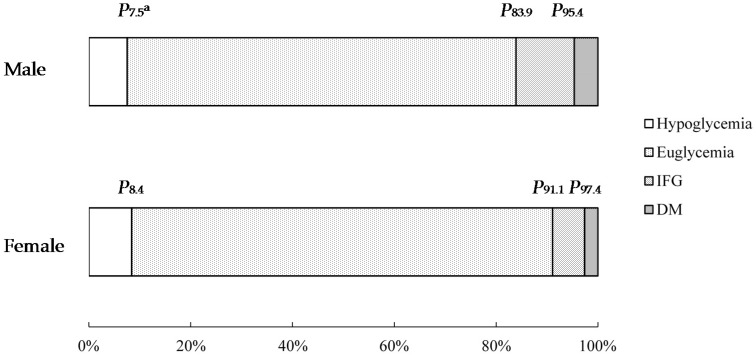
The distribution of FBG for males and females (percentage diagram). (^a^ *P*_x_ was used to represent the percentile x; IFG: impaired fasting glycaemia; DM: diabetes mellitus.).

**Figure 2:**
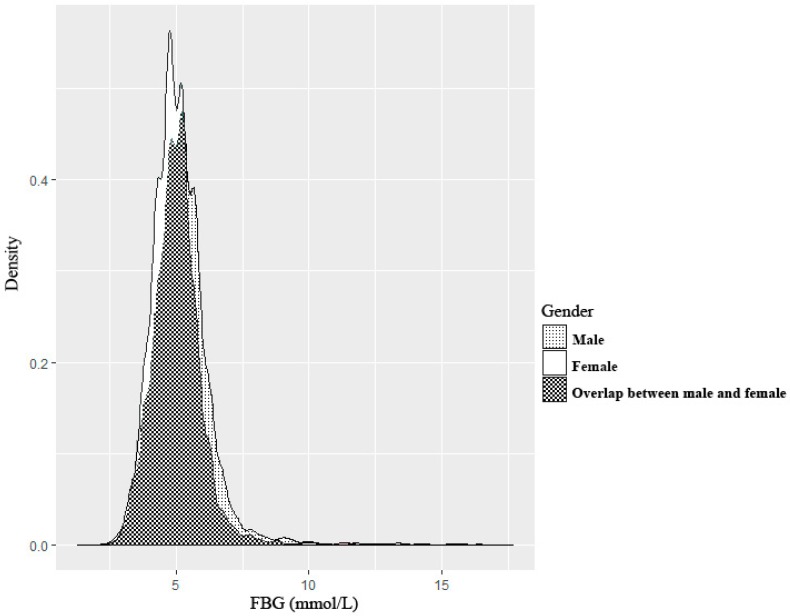
The distribution of FBG for males and females (density diagram).

Figure 1 and Figure 2 showed that the distribution of FBG was different between males and females (*χ*^2^ = 175.30, *p* < 0.001). Therefore, the following results obtained by QR model were listed separately for males and females.

Table 2 and Supplementary Figure S1 showed a relationship between the FBG and influencing factors in different quantiles for the males. BMI had a positive correlation with FBG (*P*_5_ to *P*_83.9_), however the regression coefficient had a tendency to decline. WC was positively associated with FBG in high quantiles (*P*_95.4_ to *P*_97_). Age presented a positive association with FBG in all quantiles and displayed a slight increasing trend. Similarly, TG showed a positive correlation with FBG. Living in urban areas (*P*_5_ to *P*_83.9_), drinking (*P*_5_ to *P*_83.9_), fruit intake (*P*_5_ to *P*_83.9_), family history of DM (*P*_75_ to *P*_97_), and LDL-c (*P*_25_ to *P*_97_) were positively linked with FBG.

Table 3 and Supplementary Figure S2 showed a relationship between the FBG measures and influencing factors in different quantiles for the females. BMI was positively associated with FBG in the low and middle quantiles (*P*_8.4_ to *P*_50_), and WC had a positive association with FBG in the high quantiles (*P*_50_ to *P*_97.4_). Age also showed an increasing positive correlation with FBG in all quantiles. With respect to occupation, mental labor had positive association with FBG in low and middle quantiles (*P*_5_ to *P*_95_), and manual labor was positively associated with FBG in middle and high quantiles (*P*_50_ to *P*_97.4_). Living in urban areas (*P*_5_ to *P*_75_), fruit intake (*P*_5_ to *P*_75_), meat intake (*P*_8.4_ to *P*_95_), family history of DM (*P*_25_ to *P*_91.1_), level of TG (*P*_8.4_ to *P*_97.4_), and level of TC (*P*_5_ to *P*_97.4_) were positively linked with FBG. HDL-c (*P*_5_ to *P*_95_) and fish intake (*P*_75_ to *P*_97.4_) were negatively linked with FBG.

## 4. Discussion

DM has become a major public health problem in China [23], and a number of researchers have investigated risk factors of DM [8,18,24], but little is known about the associations between FBG and influencing factors. The present study analyzed the dataset of chronic disease and risk factors among adults in Jilin Province to identify the factors which influencing different FBG populations. The QR model was performed, which could show detailed views of the effects of the influencing factors on FBG from different perspectives.

Weight control is one of the most important points of the prevention of DM. A number of studies have shown that there was a connection between DM and obesity, especially abdominal obesity [25,26,27]. Obesity is believed to decrease insulin sensitivity through damaging insulin receptors in peripheral target tissue cell membrane, eventually leading to the failure of pancreatic β-cell function [28,29]. The present study showed details of associations between BMI and FBG, which indicated that increase of BMI might elevate the levels of FBG in the non-diabetic. However, we also found that the increase in WC might have an adverse impact on FBG for DM patients. Despite weight loss in DM patients, accumulation of abdominal fat still caused a rise in FBG. Hence, it was suggested that people with normal FBG should pay attention to BMI control, and DM patients should pay attention to WC control.

Further, it has been proven that dyslipidemia was associated with blood glucose [26,30]. Our results showed that TG level was positively correlated with FBG, and displayed an increasing trend for both males and females. The possible mechanism was associated with insulin sensitivity and insulin resistance [31]. Elevated TG levels could result in higher levels of free fatty acids in serum, which would lead to obstacles in islet β-cell function [32,33]. Although the difference was not large in the mean of various serum lipid indexes between the males and females, the levels of TC, LDL-c and HDL-c showed different associations with FBG for males and females, which might be attributed to differences in sex hormones. Estrogen is commonly believed to play an important role in lipolysis [34]. It has been suggested that different prevention strategies should be adopted for different genders.

Another cohort study in China found that DM was more common in urban areas [6], and we found that living in urban places could increase level of FBG (except in the high quantiles of FBG). This may be associated with a sedentary lifestyle and dietary changes [30,35]. Plenty of epidemiological and randomized clinical studies have suggested that lifestyle interventions would have beneficial effects on FBG [36,37], such as diet control, weight control, and alcohol control, etc. We also showed that intake of alcohol elevates levels of FBG in non-diabetic participants for males, implying that the implementation of alcohol control should occur early in the normal FBG and IFG population. However, the FBG was not affected by alcohol intake in females, which may be attributed to drinking being rare among women [38].

In addition, fruit intake was positively linked to FBG in the low and middle quantiles. For now, most studies suggest increasing the intake of fruit as an important factor to reduce the risk of diabetes, with different relationships according to types and forms of consumption. Drinking fruit juice increased risk of diabetes by 15 to 24% [39], and using water instead of juice could reduce diabetes risk by 8% [40]. Therefore, the results indicated reducing the intake of juice and other elevated plasma glucose fruit would contribute to maintaining healthy FBG levels. In addition, we found intake of meat was positively associated with FBG levels in the population with euglycemia and IFG. Intake of fish was negatively associated with FBG level in the high quantile. It was suggested that using fish as a high-quality source of protein instead different kinds of meat for people with high FBG would be better.

Without any doubt, age is a well-known influencing factor for the onset of DM [41]. It was indicated that those in the high quantiles of FBG affected by age were more likely to have a poorer condition. In addition, we also found that working women were more likely to have an elevated FBG, and the possible reason was related to social engagement. Finally, family history of DM had a positive association with FBG in this study. In other words, those who had family history of DM were more likely to suffer from DM, thus, it was implied that people with family history of DM should pay more attention to FBG control.

Some limitations should be noted in present study. Firstly, participants in the study were from the Jilin Province in northeast China, which might limit the generalization of the results to other areas. Secondly, the respondents’ health-related behaviors and dietary habits were based on self-reporting, which may have bias. Thirdly, some of the variables were not quantifiable, and thus more details could not be provided. Fourthly, 36% of subjects were excluded due to missing values of FBG and/or having glycemic control, which might bias the statistical analysis in study. Finally, we did not correct the analysis for the multiple testing problem. The results need to be interpreted as explorative and should to be validated independently.

## 5. Conclusions

The effects of factors are different in different quantiles of FBG. FBG had a positive association with BMI in normal FBG, and had a positive association with WC in high FBG. Diet and alcohol intake were associated with FBG in normal FBG. FBG was more likely to be elevated in the elderly, female workers, and people with family history of DM.

## Figures and Tables

**Table 1:** Descriptive characteristic of participants by gender {mean ± SD/median [IQR]/*n* (%)}.

Variables	Male (*n* = 6734)	Female (*n* = 7964)	*t*/Z/*χ*^2^	*p*-Value
Age *	45.89 ± 13.86 46.00 [21.00]	47.40 ± 12.68 47.00 [18.00]	−6.83	<0.001
BMI *	24.23 ± 3.73 24.02 [5.05]	23.96 ± 3.63 23.67 [4.80]	4.41	<0.001
WC *	84.23 ± 10.49 84.00 [15.70]	79.56 ± 10.14 79.00 [14.00]	27.26	<0.001
FBG *	5.24 ± 1.20 5.20 [1.10]	4.98 ± 1.08 4.90 [1.00]	13.52	<0.001
TG *	2.08 ± 2.01 1.50 [1.41]	1.68 ± 1.38 1.32 [1.09]	13.54	<0.001
TC	4.89 ± 1.04 4.78 [1.29]	4.89 ± 1.08 4.76 [1.37]	−0.05	0.963
LDL-c *	2.91 ± 0.86 2.84 [1.07]	2.96 ± 0.90 2.85 [1.17]	−3.23	0.001
HDL-c *	1.37 ± 0.41 1.30 [0.46]	1.44 ± 0.37 1.40 [0.49]	−10.95	<0.001
Residence			2.45	0.118
Rural	3029 (45.11)	3685 (54.89)		
Urban	3705 (46.41)	4279 (53.59)		
Occupation *			471.45	<0.001
Unemployed/Others	1020 (29.91)	2390 (70.09)		
Mental labor	1416 (47.22)	1583 (52.78)		
Manual labor	4298 (51.85)	3991 (48.15)		
Smoking *			4126.75	<0.001
No	2287 (25.12)	6817 (74.88)		
Yes	4447 (79.50)	1147 (20.50)		
Drinking *			3848.30	<0.001
No	2828 (28.31)	7161 (71.69)		
Yes	3906 (82.95)	803 (17.05)		
Family history of DM *			22.14	<0.001
No	5944 (46.57)	6820 (53.43)		
Yes	790 (40.85)	1144 (59.15)		
Vegetable *			22.08	<0.001
Occasionally/rarely	139 (61.23)	88 (38.77)		
Often	6595 (45.57)	7876 (54.43)		
Fruit *			345.09	<0.001
Occasionally/rarely	3700 (53.98)	3154 (46.02)		
Often	3034 (38.68)	4810 (61.32)		
Meat *			442.42	<0.001
Occasionally/rarely	3731 (39.39)	5740 (60.61)		
Often	3003 (57.45)	2224 (42.55)		
Fish *			116.54	<0.001
Occasionally/rarely	5821 (44.29)	7322 (55.71)		
Often	913 (58.71)	642 (41.29)		
Eggs/Bean/Bean products *			30.44	<0.001
Occasionally/rarely	2523 (43.03)	3340 (56.97)		
Often	4211 (47.66)	4624 (52.34)		
Milk/Dairy products *			20.45	<0.001
Occasionally/rarely	5783 (46.61)	6623 (53.39)		
Often	951 (41.49)	1341 (58.51)		
Exercise *			6.34	0.012
Occasionally/rarely	4798 (45.17)	5823 (54.83)		
Often	1936 (47.49)	2141 (52.51)		

* *p* < 0.05. SD: standard deviations; BMI: body mass index; WC: waist circumference; FBG: fasting blood glucose; TG: triglyceride; TC: total cholesterol; LDL-c: low-density lipoprotein cholesterol; HDL-c: high-density lipoprotein cholesterol; IQR: inter-quartile range; DM: diabetes mellitus.

**Table 2:** Quantile regression coefficients between FBG and variables for males.

Factors	Hypoglycemia				Euglycemia						IFG		DM			
	*P*_5_		*P*_7.5_		*P*_25_		*P*_50_		*P*_75_		*P*_83.9_		*P*_95.4_		*P*_97_	
	*β*	*p*	*β*	*p*	*β*	*p*	*β*	*p*	*β*	*p*	*β*	*p*	*β*	*p*	*β*	*p*
Age	0.010	<0.001	0.008	<0.001	0.005	<0.001	0.005	<0.001	0.009	<0.001	0.011	<0.001	0.023	<0.001	0.026	<0.001
Urban	0.267	<0.001	0.269	<0.001	0.271	<0.001	0.205	<0.001	0.182	<0.001	0.185	<0.001	0.034	0.580	0.032	0.794
Drinking	0.205	<0.001	0.180	<0.001	0.089	0.002	0.094	<0.001	0.079	0.008	0.088	0.005	0.121	0.082	0.111	0.355
Fruit	0.109	0.032	0.108	0.003	0.086	0.003	0.082	0.001	0.079	0.007	0.049	0.124	0.077	0.191	0.016	0.886
Family history of DM	0.036	0.601	0.092	0.220	0.057	0.197	0.031	0.353	0.080	0.034	0.185	0.002	0.266	0.025	0.505	0.060
BMI	0.040	0.002	0.043	<0.001	0.045	<0.001	0.035	<0.001	0.026	0.002	0.019	<0.001	−0.016	0.399	−0.047	0.012
WC	−0.005	0.209	−0.005	0.115	−0.006	0.035	−0.001	0.752	0.003	0.379	0.004	0.052	0.017	0.009	0.029	0.027
TG	0.057	0.001	0.060	<0.001	0.072	<0.001	0.095	<0.001	0.152	<0.001	0.179	<0.001	0.355	<0.001	0.390	<0.001
LDL-c	−0.053	0.075	−0.028	0.222	0.044	0.018	0.042	0.011	0.052	0.007	0.060	0.005	0.101	0.018	0.136	0.034

*β*: quantile regression coefficients; IFG: impaired fasting glycaemia.

**Table 3:** Quantile regression coefficients between FBG and variables for females.

Factors	Hypoglycemia				Euglycemia						IFG				DM	
	*P*_5_		*P*_8.4_		*P*_25_		*P*_50_		*P*_75_		*P*_91.1_		*P*_95_		*P*_97.4_	
	*β*	*p*	*β*	*p*	*β*	*p*	*β*	*p*	*β*	*p*	*β*	*p*	*β*	*p*	*β*	*p*
Age	0.006	<0.001	0.006	<0.001	0.007	<0.001	0.009	<0.001	0.010	<0.001	0.014	<0.001	0.012	<0.001	0.016	0.001
Urban	0.143	<0.001	0.140	<0.001	0.134	<0.001	0.137	<0.001	0.114	<0.001	0.049	0.202	0.049	0.419	0.060	0.555
Occupation																
Unemployed/Others	-	-	-	-	-	-	-	-	-	-	-	-	-	-	-	-
Mental labor	0.118	0.018	0.119	0.014	0.045	0.252	0.063	0.043	0.099	0.005	0.107	0.036	0.116	0.042	0.249	0.079
Manual labor	0.025	0.584	0.018	0.647	0.019	0.555	0.087	<0.001	0.117	<0.001	0.112	0.015	0.145	0.022	0.290	0.017
Fruit	0.082	0.026	0.048	0.105	0.057	0.034	0.068	0.002	0.071	0.004	−0.016	0.780	−0.102	0.017	−0.054	0.616
Meat	0.043	0.239	0.061	0.046	0.044	0.119	0.058	0.015	0.096	<0.001	0.089	0.036	0.123	0.018	0.004	0.966
Fish	−0.049	0.369	0.009	0.895	−0.009	0.824	0.003	0.924	−0.082	0.047	−0.108	0.018	−0.225	<0.001	−0.384	<0.001
Family history of DM	0.008	0.875	0.059	0.228	0.080	0.037	0.082	0.003	0.091	0.006	0.149	<0.001	0.082	0.173	0.120	0.496
BMI	0.013	0.010	0.022	0.002	0.016	0.005	0.011	0.022	0.006	0.265	0.010	0.278	−0.013	0.168	−0.038	0.075
WC	0.003	0.297	0.001	0.813	0.002	0.302	0.005	0.009	0.008	<0.001	0.012	0.001	0.019	<0.001	0.030	0.002
TG	0.008	0.682	0.032	0.047	0.052	<0.001	0.078	<0.001	0.102	<0.001	0.180	<0.001	0.331	<0.001	0.500	<0.001
TC	0.069	0.001	0.053	0.002	0.044	0.003	0.042	<0.001	0.063	<0.001	0.077	0.002	0.076	0.029	0.216	0.005
HDL-c	−0.131	0.013	−0.137	0.010	−0.098	0.017	−0.113	<0.001	−0.189	<0.001	−0.170	0.011	−0.197	0.041	−0.188	0.301

## Supplementary Materials

The following are available online at www.mdpi.com/1660-4601/14/11/1368/s1, Supplementary Sampling Method. Figure S1: Quantile regression coefficients and 95% confidence intervals between FBG and variables for males. Figure S2: Quantile regression coefficients and 95% confidence intervals between FBG and variables for females.
