# Supplementary material for: Associations of Fasting Blood Glucose with Influencing Factors in Northeast China: A Quantile Regression Analysis
Source: Int J Environ Res Public Health. 2017 Nov 10;14(11):1368. doi: 10.3390/ijerph14111368 (PMC5708007; doi:10.3390/ijerph14111368)
Supplement: Supplementary file 1 [file ijerph-14-01368-s001.pdf]

Online Supplementary Material to

Associations of Fasting Blood Glucose with Influencing Factors in Northeast China: A Quantile Regression Analysis

1. Sampling Method

Five-stage stratified random cluster sampling was used to select the samples under study. In the first stage, 32 districts/counties were identified in proportion to population, geographic location and ethnicity, from nine cities (Changchun, Jilin, Siping, Liaoyuan, Tonghua, Baishan, Songyuan, Baicheng and Yanbian). At the second stage, three or four towns (depending on the size of the district) were selected by stratified random sampling to guarantee the representativeness of each sample. In the third stage, three neighborhood committees were chosen by stratified random sampling from each of the towns previously selected. In the fourth stage, one village from each chosen neighborhood committee was selected by simple random sampling. In the final stage, cluster random sampling was used to identify individuals aged 18 to 79 years old from each of the villages selected for the study.

2. Supplementary Figure 1 and 2

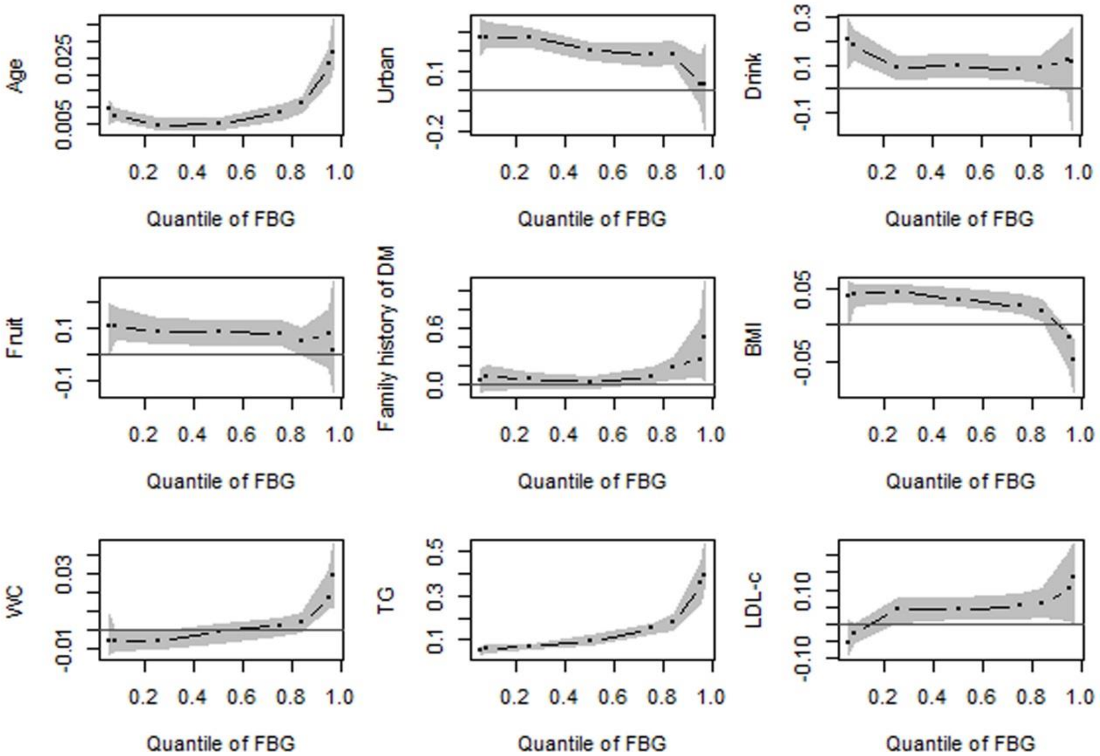

Supplementary Figure 1. Quantile regression coefficients and 95% confidence intervals between FBG and variables for males

(BMI: body mass index, WC: waist circumference, TG: triglyceride, LDL-c: low-density lipoprotein cholesterol.)

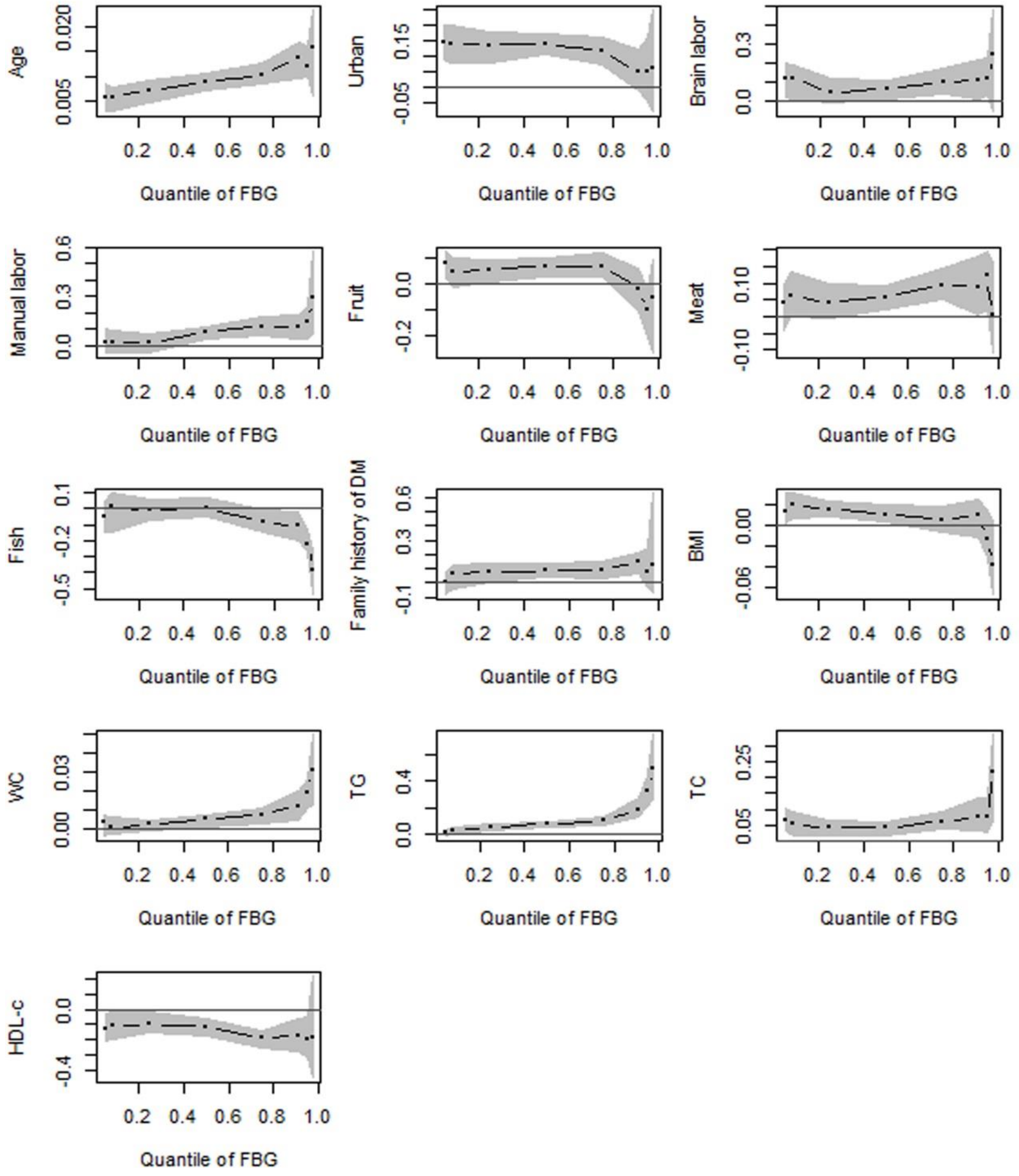

Supplementary Figure 2. Quantile regression coefficients and 95% confidence intervals between FBG and variables for females

(BMI: body mass index, WC: waist circumference, TG: triglyceride, TC: total cholesterol, HDL-c: high-density lipoprotein cholesterol.)
